# Attuning to the World: The Diachronic Constitution of the Extended Conscious Mind

**DOI:** 10.3389/fpsyg.2020.01966

**Published:** 2020-08-21

**Authors:** Michael D. Kirchhoff, Julian Kiverstein

**Affiliations:** ^1^Department of Philosophy, University of Wollongong, Wollongong, NSW, Australia; ^2^Department of Psychiatry, Amsterdam University Medical Center, University of Amsterdam, Amsterdam, Netherlands

**Keywords:** extended consciousness, extended mind, cultural practices, diachronic constitution, ultimate explanation, proximate explanation

## Abstract

It is a near consensus among materialist philosophers of mind that consciousness must somehow be constituted by internal neural processes, even if we remain unsure quite how this works. Even friends of the extended mind theory have argued that when it comes to the material substrate of conscious experience, the boundary of skin and skull is likely to prove somehow to be privileged. Such arguments have, however, typically conceived of the constitution of consciousness in synchronic terms, making a firm separation between proximate mechanisms and their ultimate causes. We argue that the processes involved in the constitution of some conscious experiences are diachronic, not synchronic. We focus on what we call phenomenal attunement in this paper—the feeling of being at home in a familiar, culturally constructed environment. Such a feeling is missing in cases of culture shock. Phenomenal attunement is a structure of our conscious experience of the world that is ubiquitous and taken for granted. We will argue that it is constituted by cycles of embodied and world-involving engagement whose dynamics are constrained by cultural practices. Thus, it follows that an essential structure of the conscious mind, the absence of which profoundly transforms conscious experience, is extended.

## Introduction

In this paper, we set out to defend the thesis of the *extended conscious mind* (ECM). We do so because we take it that the mind in general is first and foremost widely and diachronically constituted. The vast majority of what humans think and experience unfolds over time through bouts of situated engagement with the environment. This does not just hold for unconscious problem solving as many leading exponents of extended mind theory are disposed to argue. These philosophers will happily concede that some of our unconscious cognition is accomplished by cycles of perception and action in which the cognizer makes active use of resources located in the environment around them (see, e.g., Clark and Chalmers, 1998; Clark, 2008; Menary, 2010; Sutton et al., 2010; Wheeler, 2010; Kirchhoff, 2012; Kiverstein, 2018). Most of these philosophers have, however, been unwilling to generalize such arguments to consciousness (see, e.g., Chalmers, 2008, 2019; Clark, 2009, 2012). They have argued that when it comes to consciousness the boundary of the skin and skull will somehow turn out to be privileged and special. Others have conceded that in some sense ECM is possible (Wheeler, 2015). But they have claimed that specific arguments for ECM have thus far failed to make a convincing case that consciousness actually does extend. They claim that our best sciences of consciousness make it highly likely that consciousness will turn out to be a purely “in-the-head,” brain-based phenomenon (Clark, 2009; Wheeler, 2015).

We argue, by contrast, that there are no good grounds for setting up a divide between unconscious cognition and conscious perceptual experience. What is good for the goose (extended unconscious cognition) is also good for the gander (extended conscious experience). The boundary of skin and skull has no special properties such that only the processes that fall within this boundary have what it takes to support conscious experience. The cognitive agent is what Susan Hurley called a *dynamical singularity*—one that forms out of a field of causal flows, some of which loop out into the world through cycles of perception and action (Hurley, 1998). Thus, the boundary of the conscious mind can, in the right kind of circumstances, form in an agent’s dynamical coupling with its environment.

In what follows, we restrict our argument to a phenomenological structure of everyday lived experience we term “phenomenal attunement”—the feeling of being at home in a familiar culturally constructed environment. This phenomenological structure forms in the co-constituting coupling of the human agent with its social and cultural environment. We talk of the “co-constitution” of agent and environment because we will argue both agent and environment form together. The individual’s cognitive capacities are partially constituted by environmental structures, practices, and institutions. At the same time, these structures, practices and institutions are the product of human cultural activities. There is no end point in the process leading to the experience of phenomenal attunement after which the individual can throw away the cultural environment and rely solely upon the brain. Since phenomenal attunement is a structure of conscious experience, this will provide us with an argument for why the person’s conscious experience cannot always be generated solely out of processes unfolding inside the person’s brain, uncoupled from the surrounding environment. The cultural environment plays a constituting role because we get to experience only phenomenal attunement (and its corollary of phenomenal disattunement) in our ongoing co-constituting coupling with a social and culturally constructed niche.

Internalist critics of ECM will be quick to insist (mistakenly, we believe) that internalism is entirely consistent with this line of argument. They will most likely object that the brain is causally dependent on specific forms of agent-environment couplings to settle on the pattern of neural activity constitutive of a particular conscious experience (see, e.g., Adams and Aizawa, 2001; Rupert, 2009). Internalists will concede that the world plays an ongoing *causal* role in driving the brain into a certain neural configuration allowing for the emergence of conscious experience. But they will insist it is the particular neural configuration in question that materially constitutes the conscious experience (see also Clark, 2009; Wheeler, 2015). They will thus take issue with our talk of co-constituting coupling of agent and environment.

We address this objection directly by arguing that it rests on a misunderstanding of the distinction between causation and constitution, treating one as strictly diachronic (causation) and the other as wholly synchronic (constitution). The constitution relation is generally cast as a strictly non-causal (i.e., atemporal/synchronic) one of dependence. We argue (building on Kirchhoff, 2015b and Kirchhoff and Kiverstein, 2019a) such an understanding of constitution while appropriate for material objects, is ill-suited when it comes to characterizing dynamic and processual phenomena such as conscious experience. To adequately characterize the constitution of a process, it is both possible and fruitful to understand the concept of constitution as a diachronic relation of dependence. The notion of diachronic constitution we argue leads naturally to an extended account of phenomenal attunement, incorporating both ultimate and proximate causes.^1^

The structure of the paper is as follows. In section “The Diachronic Constitution of Phenomenal Attunement: The Case of Culture Shock,” we start by explaining what we mean by phenomenal attunement. We illustrate this phenomenon by reference to the cases of culture shock and psychopathology in which it is disturbed. We argue that culture shock shows how the experience of being attuned to the cultural environment is an integral part of the phenomenology of our everyday conscious experiences. But phenomenal attunement is also constitutively dependent on the ongoing coupling of an individual to her cultural environment through cycles of perception and action. Thus, phenomenal attunement provides us with a case that illustrates how coupling to the cultural environment diachronically constitutes a core dimension of conscious experience. Section “Assembling the Mind: Cognitive Assembly and The Pac-Man Intuition” takes up a likely internalist objection to our argument. Arguments for the extended mind have tended to limit bouts of extended cognition to short, synchronic timescales. We argue that this focus on the synchronic is problematic, as it precludes dynamical processes unfolding over longer periods of time, from being more than ultimate (background) causes against which the brain assembles the elements that make up extended minds. We propose a new metaphysics of constitution, cast in terms of diachronic constitution that avoids this consequence. In section “Synchronic and Diachronic Constitution,” we review the standard notion of constitution (synchronic constitution), which we then contrast with the diachronic conception of constitution required for understanding the constitution of dynamic processes. We suggest that the diachronic conception of constitution is required to account for the metaphysics of extended minds. This is because extended cognition is dynamic, unfolding over time through cycles of situated engagement with the affordances or possibilities for action the environment furnishes (Anderson et al., 2012; Kiverstein, 2018). In section “Objections: Pluggability Intuitions, Free-Floating Brains and Internal Fantasies,” we provide responses to three objections against our argument for ECM. These objections aim to defend the consensus view among materialist philosophers of mind that all experiences must be somehow constituted out of internal neuronal processing. In section “Wide and Diachronic Constitution: Two Conceptual Flips,” we tackle the often made objection that arguments for the extended mind (EM) are guilty of conflating causal coupling with the metaphysical relation of constitution. We argue that once one makes the turn to diachronic constitution, this objection against EM, and by extension ECM, loses its force. We end section “Wide and Diachronic Constitution: Two Conceptual Flips,” by showing how the diachronic view of constitution we argue for in this paper can safely avoid the cognitive bloat objection often raised against EM (see, e.g., Rowlands, 2009; Sprevak, 2009).

## The Diachronic Constitution of Phenomenal Attunement: The Case of Culture Shock

As an illustration of what we mean by phenomenal attunement, we will begin by considering the example of culture shock. An experience of culture shock is characterized by feelings of distress and alienation. These feelings of distress and alienation are examples of an absence of phenomenal attunement with the cultural environment. A much-discussed case is 13-year-old Eva Hoffman, who, along with her mother and father, left Poland in 1956 for the prospects of a better life in Vancouver, Canada. Even though Eva had her parents by her side, her experiential world changed dramatically. She explains:

[T]he country of my childhood lives within me with a primacy that is a form of love …. It has fed me language, perceptions, sounds…. It has given me the colors and the furrows of reality, my first loves (Hoffman, 1989, pp. 74–5; quoted in Wexler, 2008, p. 175).

Having spent only three nights in Vancouver, she reports waking up from a dream, wondering:

[W]hat has happened to me in this new world? I don’t know. I don’t see what I’ve seen, don’t comprehend what’s in front of me. I’m not filled with language anymore, and I have only a memory of fullness to anguish me with the knowledge that, in this dark and empty state, I don’t really exist (Hoffman, 1989, p. 180; quoted in Wexler, 2008, p. 175).

Culture shock illustrates how expectations that have their origin outside of the individual in patterns of cultural practices attune us to a shared cultural environment. Should the individual move to a new environment, the result may be misalignment and pervasive, hard-to-suppress violation of her expectations about her shared social and cultural environment. We will argue that to properly explain cases such as culture shock we need to appeal to an extended dynamic singularity comprising Eva’s internal neurobiological states, the patterns of practice that are enacted within her cultural niche, and her sensory and active states that couple her to her cultural environment. To explain her current experiences one must take into account the expectations that she has formed through her past involvement in cultural practices and the role of these expectations in shaping the phenomenology of her ongoing experience. It is not just her past that we need to take into account but also her present circumstances and her orientation to the future in her new cultural environment.

Phenomenal attunement can be formalized as the divergence between prior expectations about the causes of sensory observations and the actual causes (e.g., generated via patterns of cultural practice). The experience of phenomenal attunement can be described as the Kullback–Leibler divergence between prior expectations (*P*^∗^) and cultural practices (*P*o) generating sensory states: *C*_exp_ = *D*_KL_ [*P*^∗^ || *P*o].^2^ Phenomenal attunement comes in degrees and varies with divergence between *P*^∗^ and *P*o. There is an experience of phenomenal lack of attunement when *D*_KL_ [*P*^∗^ || *P*o] > 0. On average, one would expect expectations to converge on cultural practices, ensuring phenomenal attunement to one’s cultural environment. Suppose, now, that we associate experiences of culture shock (feelings of distress and alienation) with uncertainty; then the higher the divergence between *P*^∗^ and *P*o, the more uncertainty is expressed in the coupling between *P*^∗^ and *P*o. Crucially, if there is high uncertainty as a consequence of the divergence between *P*^∗^ and *P*o, the subject will need to exert more effort to make sense of her surroundings. If one’s expectations systematically fail to align with the regularities (causal and statistical) of one’s environment, feelings of distress are likely to arise as one needs to make much more effort to make sense of how one finds oneself situated in the world.

We are all familiar with such situations, where uncertainty about outcomes of social interactions yield sensations of frustration or discontent. Alignment and continued attunement to other people and to wider patterns of practice are integral parts of the phenomenology of our everyday conscious experiences. Slaby (2016) invites us to imagine working as an intern in a large company:

Your first days working in the firm will be marked by experiences like the following: You find the regular employees speaking, acting, moving, and comporting themselves in ways that are unfamiliar to you in various ways. Not only will their work routines be new to you, but also their styles of interacting, of comporting themselves, of resonating affectively with one another, the ways of address, of conversing with superiors, the use of humor to begin a conversation, or deflate a moment of tension, when and how to display certain feelings openly (enthusiasm maybe, or pride after an achievement), or suppressing others (no fear, no insecurities), and so on (Slaby, 2016, p. 1).

As an intern you initially experience a phenomenal lack of attunement to others in the workplace. So much of what takes place between colleagues in a large corporation is the enactment of a past history of interaction to which outsiders are not privy. To align and adjust to this novel niche, an intern will need to become familiar with what other employees take for granted. She must learn more than how to perform her work routine. She must cultivate a sense of how to interact with her colleagues and what is at issue in these interactions. As long as she does not have a sense of this, she will experience just the same or similar feelings of distress and alienation associated with culture shock. Perception and action in social domains such as a large corporation are organized by norm-regulated practices—regular, stable, and ordered patterns of activity. Norms structure social interactions within a social domain. One must become attuned to the norms that govern interactions in domains of social life in order to feel at home in these domains of social life.

Thus, maintaining attunement with one’s cultural surroundings critically depends on a match in an individual’s expectations and the normatively regulated expectations of other participants in a social practice (Kirchhoff and Kiverstein, 2019a, ch. 5).^3^ The expectations that guide one’s perception and action must match the expectations that form in patterns of practice. Pervasive and sustained lack of attunement can prove to be pathological. People suffering from schizophrenic delusion have been hypothesized to have a high expectation of noise and uncertainty. They expect more sensory noise than there really is in a given context with the consequence that they are unable to find the signal among the noise. This leads them to neglect sensory evidence in favor of their prior expectations (Fletcher and Frith, 2009; Hohwy, 2015). The effect of this aberrant weighting of evidence and general failure of context-sensitive updating of prior expectations is that they come to inhabit a delusional reality that is increasingly cut off and removed from the common-sense, everyday, familiar reality they share with other people (Sass, 1994). They increasingly come to inhabit their own solipsistic reality. People with autism by contrast give too much weight to new sensory evidence. This weighting of sensory evidence leads to sensory information that conflicts with their prior expectations to dominate in processing, which has the consequence that they have difficulties in becoming attuned to more stable and persistent regularities (Pellicano and Burr, 2012; Lawson et al., 2014; Palmer et al., 2017). The consequence of this aberrant weighting of sensory information in both cases is that people have difficulties in becoming phenomenally attuned to non-autistic cultural practices.^4^

In culture shock this attunement to the everyday world is also temporarily lost, and this leads to a deep disturbance of lived experience with the divergence between *P*^∗^ and *P*o being high. Crucially, attunement, as well as lack of attunement, relies on the ongoing coupling of Eva to the cultural world through cycles of perception and action. Phenomenal attunement is, as we have suggested, the outcome of synchronous coupling between internal and external states mediated via sensory and active states (c.f. Kirchhoff and Kiverstein, 2019a). The person is coupled to her cultural environment by sensory and active states. Patterns of practice structure what we expect to experience in our cultural environment. Repeated engagement in these practices establishes the norms and rules of conduct that push back should one deviate from them. Think of Slaby’s example of the workplace practices and patterns of micro-interaction in a large corporation. Roepstorff et al. (2012) state: “Culture gets under the skin and skull, … and it is remade gradually through collective instances of actualization” (p. 1052). This normatively regulated coupling plays a constituting role in the generation of conscious experience of phenomenal attunement or lack of attunement.

Phenomenal attunement is not constituted synchronically by underlying brain states at a snapshot instant in time. If conscious experience is equal to the Kullback–Leibler divergence between prior expectations (*P*^∗^) and cultural practices (*P*o) generating sensory states, *C*_exp_ = *D*_KL_ [*P*^∗^ || *P*o], then attunement cannot be constituted by the proximate mechanisms involved in generating *P*^∗^. The KL-divergence is a *relational measure* of the distance between *P*^∗^ and *P*o. An individual agent’s coupling to a culturally constructed environment is best understood diachronically, not synchronically. The individual’s activities are constrained by the norms that govern the regular and ordered patterns of activities that stabilize in a community over time. Thus, the experience of phenomenal lack of attunement that arises from failing to become adequately attuned to the cultural environment is best understood in terms of dynamical processes that unfold over multiple interacting timescales. In other words, it is best understood diachronically. Let us unpack this argument step by step.

We have analyzed Eva’s experience of culture shock in terms of expectations *P*^∗^ formed out of her involvement in past practices associated with her childhood in Warsaw that fail to align with the cultural environment she now inhabits in Vancouver. The cycles of perception and action that couple her to her cultural surroundings are “permeated” and “infused” by her expectations (Gallagher, 2018; Hutto et al., 2019). Her expectations provide her with a background understanding of her surroundings in virtue of which she encounters a familiar environment in which she knows how to act. Think again of our example of the office intern who is yet to be initiated into the styles of interacting taken for granted by other employees. Eva’s expectations, however, fail to align with the normative expectations regulative of people’s activity in Vancouver. It is these expectations that she brings to bear to make sense of her present situation and to orient how she engages with her surroundings in the future. However, they fail to orient her adequately to the normative expectations operative in her current environment. The result of this lack of alignment is her experience of phenomenal lack of attunement.

Those in the grip of internalist intuitions might agree with us that patterns of cultural practices are involved in setting the parameters of brain-based processes over long (ultimate) timescales. Yet they will insist that in the here and now, conscious experience is determined entirely by proximate mechanisms in the brain unconsciously inferring hypotheses about the hidden external causes of sensory data. To insist otherwise would be to fallaciously confuse ultimate causes with proximate ones in an explanation of consciousness. Our objector might attempt to bolster such intuitions by invoking neural duplicates. Would Eva’s neural duplicate in the present moment not have the same phenomenal experience as Eva just now? This objection turns on the idea that it is the neural machinery and the particular forms of information processing it supports that do the work of constituting the conscious mind synchronically, not a history of interaction with the environment. We show why we think such an objection is misplaced in the next section.

## Assembling the Mind: Cognitive Assembly and the Pac-Man Intuition

The debate about the extended mind (EM) really took off in the philosophy of mind with the publication of a short paper in *Analysis* by Clark and Chalmers (1998). The aim of the paper was to invite readers to question the (biological chauvinist) assumption that anything located external to skin and skull cannot be a part of a person’s mind. Clark and Chalmers (1998) devised much discussed thought experiments aimed at showing how something is a part of a person’s mind because of the causal role it plays in guiding the person’s behavior. Artifacts, such as notebooks located outside of the individual’s body, can become fully integrated parts of an individual’s thinking processes. By coupling with tools and technologies the individual can accomplish her thinking and problem solving. Thus, artifacts can form a part of a larger cognitive system the individual relies upon in acting. They can come to play a constitutive role in the production of the individual’s behavior equivalent to that played by processes internal to the individual. Clark and Chalmers (1998) argued that we should not exclude the things around us from counting as parts of our minds simply because of their location outside of the head; rather, if external elements play the right kind of functional role in driving cognitive processes, such elements should count as part of someone’s mind—just as internal states playing such roles would naturally qualify as part of one’s mental machinery.

Clark in his later work was up-front about giving a privileged place to the agent in the assembly or formation of extended cognitive processes (Clark, 2008). He writes: “Human cognitive processing (sometimes) literally extends into the environment surrounding the organism. But the organism (and within the organism, the brain/CNS) remains the core and currently the most active element” (p. 139). The individual cognizer decides, in part based on efficiency considerations, whether to rely solely on her own on-board (neural) cognitive machinery to solve a problem or to softly assemble a solution that makes use of resources located in the external environment. The work of assembling a cognitive system that can solve a particular problem is delegated to the brain of the individual. Problem solving may sometimes constitutively involve bouts of situated, real-world action that unfolds over relatively short timescales of hundreds of milliseconds, or seconds, and is orchestrated from inside of the brain of the individual. Insofar as Clark takes cognition to be organism-centered, he must insist upon a strict separation of events as they unfold over short timescales from events as they unfold in cultural practice over longer historical timescales. But many examples of situated action in the literature are examples of actions the person has learned to perform by taking part in cultural practices (Hutchins, 2011). Whereas Clark will argue it is the brain that does the work of assembling and organizing the cognitive system in these cases, we would argue (taking our lead from the cognitive anthropologist Ed Hutchins) that in many cases cultural practices organize the action in situated action and therefore in cognitive assembly.

The patterns of perception and action belong to a cultural practice because the understanding of what to say and what to do derives from rules, evaluative standards, principles, and imperatives that are operative in practice. Practices organize what people do in the sense that the tasks and projects people undertake and the purposes and ends for which people act have their origin in practices (Schatzki, 1996). What the members of a practice say and do follows from and aligns with the practice. Think again of Slaby’s example of the intern working in a large corporation. Employees are trained to think, feel, and act so that they can become attuned to playing a specific role within the corporate machine. People already habituated to working in the company will reinforce and sanction or punish what the intern says and does in more or less subtle ways until what she says and does is well aligned with the prevalent styles of interaction in this institution. Individuals are situated in practices, but the practices also situate what individuals do and say. Clark and Chalmers (1998) put forward the hypothesis of the extended mind starting from a picture in which a pre-existing individual agent occasionally connects with the world to solve a problem that it would be much harder to solve without the use of some artifact, tool, or technology. We are arguing for a view of the extended mind in which the activities of the individual agent and the agent’s cultural environment are quite literally co-constituting. The individual isn’t already fully formed but what the individual says and does is profoundly shaped and transformed by the practices they take part in.

Clark is willing to allow that cultural practices may do some of the work of *setting the scene* for the assembly and orchestration of extended cognitive processes. However, he stops short of allowing cultural practices to form a part of the slow unfolding processes out of which extended minds form. Clark concedes what no doubt everyone will allow—that a child must have learned to read and write before she can make use of pen and paper to do multiplication. However, he argues that when the child makes use of the external scaffolding of pen and paper to do long multiplication, she does so over relatively short time scales. But why does Clark privilege processes unfolding in the synchronic here and now? What the child is doing in making use of pen and paper is reenacting what she has learned by taking part in a practice. The actions she performs are embedded in and organized by the practice of which she is a part. History and culture are always embedded as well as carried along in the practices and artifacts individuals are engaging with (Menary, 2007, 2010; Sutton, 2010; see also Haugeland, 2002). The result of focusing only on the synchronic timescale—i.e., on proximate causes—is that everything that makes a difference outside of the here and now must be treated at best as making a causal contribution to mentality, either as background conditions or as input to internal neural processes.

Clark’s reasoning has the problematic consequence that minds must be fully constituted over short-term timescales.^5^ History and culture form background conditions that set the stage for the brain to do the real work of constituting the mind in the here and now. This wrongly assumes that all of the work of cultural practices in constraining, coordinating, and self-organizing action can come to be fully internalized. Clark seems to assume that what is learned from others through training in social practices can simply be internalized in the form of internal representations. This training can then get to do its work through its internal representation by the individual. The cultural transmission of knowledge and practices is understood as transmission of information among individuals. Once the information has circulated in the right way among individuals, there is no longer any work left for cultural practices to do.

We think this is the wrong model of how cultural learning works. To see what is mistaken in this picture, consider by way of analogy an individual we will call Pac-Man, named after the character in the arcade game. Evolution has set up Pac-Man so that on average and over time he distills the regularities of his niche. He “eats” up such regularities and comes to embody them in an internal model of his external environment. Pac-Man moves about his environment, extracting and consuming statistical structures to build up a detailed internal model of his local environment. This is how he learns about his niche. The body of Pac-Man and the wider niche in which he is situated are ultimately important for acquiring and updating the parameters of his internal model. Yet once these parameters are acquired, Pac-Man can rely on his internally encoded model of his world to act adaptively in his environment. He has consumed all of the information he needs. We will call this the Pac-Man intuition.

The Pac-Man intuition is false. We suggest by contrast that extended minds are constituted by temporally unfolding processes, and thus the Pac-Man intuition provides the wrong model for thinking about the internalization of cultural forms of knowledge. Internal models as they are embodied in living beings are tasked with always having to maintain a grip on the fluctuations in the dynamics of their local environments. The fluctuations do not reside or disappear but are constantly forming and reforming, even if in only slightly different ways. Organisms must therefore constantly *attune* their internal dynamics to the continuously changing dynamics of the environment in which they are situated. But now it might be objected this attunement takes place in the here and now. Past learning sets up dispositions to act in ways that conform with a practice. Think again of the child learning to do long multiplication. These dispositions are fully internalized. Everything that is required for the disposition to be realized in action happens synchronically in interaction with the environment and with other people, such as teachers. But to say that a disposition is internalized is not at all the same as saying that what people know when they take part in cultural practices is fully internalized. Thus, one can think of the enactment of cultural practices as happening synchronically without relying on the Pac-Man intuition to account for cultural learning.^6^

In reply, we suggest that this synchronic account of the enactment of cultural practices misses an important feature of situated action. It misses how the person’s dispositions are constrained by rules, norms, principles, and standards that operate at the scale of the cultural practice. Situated action is constituted by processes that unfold over two timescales. First, there is the timescale of the cycles of perception and action that couple the agent to the environment as in the classic example of using pen and paper to do mental arithmetic. Cycles of perception and action unfold over time and thus cannot be synchronically constituted at a time *t*. As dynamical processes, they are diachronically constituted. Second, there is the slower timescale of the cultural practices the child is initiated into in learning to do long multiplication. The dispositions the child puts into action in the here and now are constrained by what people have done over the longer period of time during which the practice of doing multiplication has taken shape and developed. It is these timescales that get out of sync with each other in cases of phenomenal attunement as was argued at the end of the previous section. The expectations that are formative for Eva due to her growing up in Poland do not align with those that are operative in her new home of Vancouver. Thus, the expectations that shape her perception and action are out of step with those of her surroundings.

One can think of this entanglement of the slower and faster timescales by comparison with the dynamics of self-organizing systems—disordered systems in which global order can arise under the influence of the system’s own dynamics. We observe the emergence of global order in such systems when a control parameter reaches a critical value that makes possible new forms of organization. Consider, for instance, the example of the Bénard effect from non-equilibrium fluid dynamics. A Bénard or convection roll forms when a fluid (for example, oil) is heated from below. The temperature difference between the surface and the bottom of the fluid is the control parameter. Once this temperature gradient reaches a critical value with more energy being introduced into the fluid than can be dissipated, the fluid becomes unstable. This instability leads to the formation of rolling, convection patterns in the oil. These rolling patterns are macroscopic states of the fluid that slowly form in the oil as it is heated. Such a macroscopic state is formed by the molecules of which the oil is composed. Thus, there is a constraint that runs from the micro- to the macro-scale. But crucially, the constraints also run in the other direction from the macro- to the micro-scale. When the order parameter reaches a critical value, the system enters an unstable state that allows for the convection rolls to arise. The dynamics evolving over longer timescales—the temperature gradient over the ensemble—entrains the dynamics evolving over shorter timescales—the molecules and the dissipation of energy by the fluid.

We are suggesting just the same circular causal dynamic obtains in the case of situated action. The cycles of perception and action that form over relatively short timescales can be compared to the microscopic interactions that take place in the fluid when it is heated. We are suggesting that the rules, principles, and standards—the patterns of cultural practice—can be thought of as macroscopic-order parameters that evolve over longer timescales. These patterns of practice as order parameters form out of the interactions of individuals over time. But crucially, they also entrain what individuals do over faster timescales. The cycles of perception and action that couple the individual to the cultural environment and the patterns of practice that are up and running in the cultural environment mutually constrain each other. They form a circular causal relationship.

To attempt to account for situated action synchronically, just in terms of what happens here and now, is mistaken on two grounds. It ignores how the coupling of the agent to the environment in perception and action is a dynamic process that unfolds over multiple interacting timescales. Second, it abstracts away from the wider pattern of practice that is a constraint on the situated actions people perform over shorter timescales. The cultural “training wheels” cannot, always and necessarily, be dispensed with as the Pac-Man intuition implies. This is to assume, as Hurley (2010) has pointed out, “that extended tuning and maintenance processes” are no part of the sought-for explanation of the workings of the mind (Hurley, 2010, p. 142). We’ve argued against such an assumption. Once the Pac-Man intuition is rejected, however, we will need a different account of constitution from the one that assumes the mind can be constituted at a synchronic instant in time. We need a diachronic concept of constitution.

## Synchronic and Diachronic Constitution

To introduce and develop the distinction between synchronic and diachronic constitution, a useful starting point is to get clear about the notion of a metaphysical grounding relation, of which the concept of constitution is one example. What characterizes a metaphysical grounding relation is the idea that for a relation, *R*, to qualify as a metaphysical grounding relation, *R* must express the form “*X* (or the *X*s) metaphysically determines *Y*,” when it is *by virtue of* X (or the *X*s) that *Y* exists. Thus, in the context of our paper, *Y* is the experience of phenomenal attunement or its absence in cases of culture shock. We have been arguing that phenomenal attunement is constituted by cycles of perception and action that couple the perceiver to their local cultural environment. Thus, we are claiming that it is by virtue of the person’s coupling to the cultural environment that the person has an experience of phenomenal attunement.

This *by-virtue-of* relation is often specified as a species of determination (cf. Kim, 1990; Shapiro, 2004; Polger, 2010). Different relations—such as composition, realization, and supervenience—have also been used in philosophy to express the view that *Y* exists by virtue of *X* (Kim, 1998; Bennett, 2011).

It is widely agreed that a necessary condition for *X* (or the *X*s) to constitute *Y* is that the relation of constitution that holds between X (or the Xs) and Y is a *synchronic* one-to-one, or many-to-one, relation of determination between spatially and materially co-located objects (or processes) of different kinds. A central reason for conceiving of constitution as a synchronic dependence relation is nicely articulated by Bennett: “building [grounding] relations do not unfold over time …. Causation, in contrast, is paradigmatically diachronic, and that idea is frequently invoked to distinguish causation from relations such as composition, constitution, supervenience …” (Bennett, 2011, pp. 93–94). The assumption that constitution must be a synchronic dependence relation is engrained in the very manner in which this grounding relation is analyzed. For example, it is a standard assumption on the part of constitution theorists that constitution requires spatial and material coincidence—X constitutes Y at t only if X and Y have the same spatial location at a particular time *t* and share the same material parts at that specific time *t*. It is thus presupposed that the constitution relation holds instantaneously between X (or the Xs) and Y and therefore cannot be a temporally unfolding relation. Causation, by contrast, may be said to hold between independent events or processes, in the sense that depending on the time interval between cause and effect, it is prima facie possible to think of cause as preceding its effect in time thus as occurring non-simultaneously. The standard formulation of constitution is thus that constitution is a synchronic relation of dependence.

It is not difficult to provide textual evidence for the claim that EM is typically taken to be a thesis about the constitution of minds that assumes this standard formulation of constitution (or, some other kind of metaphysical grounding relation):

*EM* is a claim about the composition or *constitution* of (some) mental processes (Rowlands, 2009, p. 54; italics added).

What is at issue, as far as the claims about cognitive extension are concerned, is simply which bits of the world make true (by serving as the local mechanistic supervenience base for) certain claims about a subject’s *here-and-now* mental states or cognitive processing (Clark, 2008, p. 118; italics added).

*Causal dependency of mentality on external factors*—even when that causal dependency is of the “necessary” kind […]—*is simply not enough* for genuine cognitive extension. What is needed is constitutive dependence of mentality on external factors, the sort of dependence indicated by talk of the beyond-the-skin factors themselves rightly being accorded fully paid-up cognitive status (Wheeler, 2010, p. 246; italics added).

As a final example consider how Shapiro characterizes the difference between causation and constitution: “[If] *C* is a constituent of an event or process *P*, *C* exists where and when that event or process exists. Thus, for some process *P*, if *C* takes place prior to *P*’s occurrence […], or if *C* takes place apart from *P*’s occurrence […], then *C* is not a constituent of P” (Shapiro, 2011, p. 160).

The metaphysics of the extended mind has thus taken for granted that constitution is a synchronic relation of determination. But is this assumption warranted? Synchronic relations are not well suited for understanding dynamical processes or their nested or hierarchical organization. But candidates for cases of extended cognition typically involve reciprocal coupling of the agent and its environment. More formally, the equations describing the behavior of the agent over time cannot be solved independently of the equations describing the environment and vice versa (Lamb and Chemero, 2018). The variables in the respective equations describe how the components of the agent and environment change in relation to each other. The equations describing change in the environment contain variables whose values correspond to changes in the agent. Conversely the equations describing change in the agent contain variables whose values correspond to changes in the environment (Anderson et al., 2012). The state changes of the agent will be dampened and amplified by state changes in the environment and vice versa. The solution to these equations is thus interdependent.

Diachronic constitution captures the basic idea that for a process to be what it is, it must unfold over time. In other words, there is no such thing as a process at an instant or synchronic point in time. For example, one often reads that water is constituted by or composed of H_2_0. This assumption is a practical assumption to make, in science as in everyday life. But it should not be taken as evidence for the further claim that water is constituted by H_2_0 at a synchronic point in time. Instead water is constituted by “oxygen and hydrogen in various polymeric forms, such as (H_2_O)_2_, (H_2_O)_3_, and so on, that are *constantly forming*, *dissipating*, and *reforming* over short time periods in such a way as to give rise to the familiar properties of the macroscopic kind water” (Ladyman and Ross, 2007, p. 21; italics added). Hence, it makes “no sense to imagine it [water] having its familiar properties synchronically” (Ross and Ladyman, 2010, p. 160). Spivey (2007) makes the exact same point in his book-length treatment of cognitive processes and their underlying mechanisms.

**FIGURE 1:**
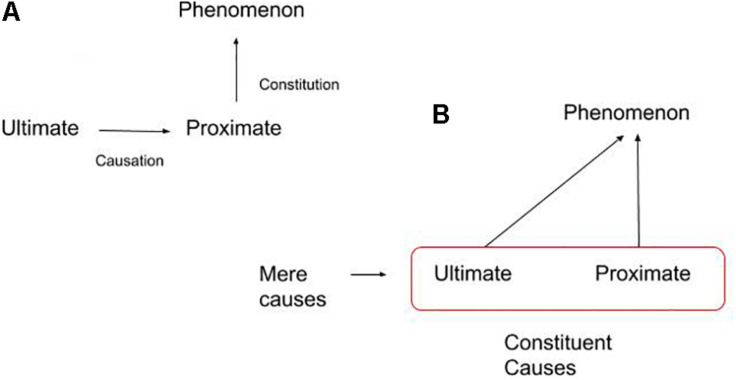
In panel **(A)**, we depict the standard way of casting the difference between causation (ultimate causes) and constitution (proximate mechanisms), where ultimate causes influence but do not constitute the phenomenon of interest. Panel **(B)** illustrates the notion of diachronic constitution, where the constituent causes for some phenomenon include ultimate and proximate causes over time, but not mere (background) causes.

We suggest that conceiving of constitution as a diachronic relation that unfolds over time makes a better fit with the extended mind in which a person’s mental states form in the dynamic coupling of the agent with its surroundings. Diachronic constitution questions a basic assumption of synchronic constitution that ultimate causes must be treated as wholly distinct from proximate constituents. For example, we can ask why birds migrate, and we can ask how they migrate. The former question can be answered by reference to the evolutionary and developmental history of birds. The latter *how*-question is answered by reference to muscle mass, morphology, and so on, *at a specific point in time*.^7^ So there is a clear temporal difference between these two forms of explanation: the ultimate explanation is a diachronic mode of explanation; whereas the proximate explanation, it might be thought, is a type of synchronic explanation. In other words, proximate *how*-explanations are mechanistic and immediate, while ultimate *why*-explanations are causal and historical. Diachronic constitution implies that the choice between ultimate and proximate explanations is sometimes a false choice. In the case of dynamical phenomena, proximate explanations will often include ultimate causes. The constitutive basis of a dynamical process or event will include both proximate mechanisms and processes unfolding over longer timescales (Figure 1).

Our claim that diachronic constitution integrates ultimate and proximate causes seems to obscure the distinction between causation and constitution. Must there always be a distinct way of identifying causes and constituents? We think not. A common strategy by which to identify constituents for specific phenomena is by determining what plays the most salient *causal role(s)* with regards to the constitution of some phenomenon. So the relevant distinction is not between causation and constitution, *per se*; rather, it is between *mere causes* and *constituent causes* (Figure 1B). Diachronic constitution as an account of the constitution of dynamic phenomena will include ultimate causes among its constituent causes.^8^

We conclude then that conceiving of constitution in diachronic terms provides the best metaphysical tools for understanding the kind of temporally nested structure that dynamical systems and processes exhibit (c.f. Kirchhoff, 2015a,c). Extended cognitive processes are constituted by many different subprocesses, each of which unfolds continuously over time exhibiting its own rate of change, rhythm and duration. Each process will be both influencing, and influenced by, the other subprocesses of which it is composed (van Gelder and Port, 1995). The constituent subprocesses may partially overlap in time but in order to contribute to the constitution of a system *S* it is not necessary that their existence entirely overlaps with that of *S*. The subprocesses that make up the agent-environment system do so over a temporally extended interval, and not at discrete instants in a stepwise and linear manner. In the remainder of our paper we make use of the notion of diachronic constitution to develop and defend an extended account of phenomenal attunement.

## Objections: Pluggability Intuitions, Free-Floating Brains, and Internal Fantasies

Consider the following twin case involving Eva and Eva^∗^. Eva and Eva^∗^ are neural duplicates. Eva, situated in Vancouver, is experiencing culture shock. Given that Eva^∗^ shares an identical neural profile with Eva, does it follow that she must also experience culture shock? We agree with Hurley (2010) that if a brain is not hooked up and plugged into an environment just like mine, it will not always be possible for this brain to play its role in generating experiences just like mine (c.f. Nöe, 2006). It is not always possible to “unplug” the internal neural factors that bring about experiences from the environment and “replug” them into a different environment without this replugging changing the functioning of the internal neural factors (Hurley, 2010). We’ll argue that phenomenal attunement counts as a case in which pluggability fails. For Eva and Eva^∗^ to be neural duplicates they must also be environmental duplicates. It is not their being neural duplicates that minimally suffices to make them phenomenal duplicates.^9^ The minimally sufficient conditions for Eva and Eva^∗^ to be phenomenal duplicates will include the cultural environment they couple to in perception and action and the normative expectations that operate this environment. This is because it is the coupling of Eva to the cultural environment and the role of culture-specific expectations in mediating this coupling that constitute her experience of culture shock. The mere notion that Eva and Eva^∗^ are neural duplicates is not by itself sufficient to make them phenomenal duplicates. To share an experience of phenomenal lack of attunement, they must be duplicate extended dynamic singularities.

To see this last point, imagine a scenario in which Eva and Eva^∗^ share identical neural states. They are synchronically identical in terms of the configuration of their brains. Eva, however, is living in her home country, Poland, and Eva^∗^ has left to take up a new life in Vancouver. Note that it would be Eva^∗^, and not Eva, who experiences culture shock. What can we appeal to for an explanation of this difference in experience? The key difference is Eva’s coupling to her local cultural environment. It is the coupling that is being intervened in in this scenario. Thus, it is Eva’s relation to her cultural environment that makes the real difference in accounting for why Eva and Eva^∗^ could be neural duplicates and still differ in their phenomenal experience. Eva^∗^ experiences a lack of attunement with her cultural surroundings because the expectations that underlie her perception and action do not match those that are operative in her local cultural environment.

We take this thought experiment to show that pluggability fails at least for the case of phenomenal attunement. One cannot hold the internal states of the agent constant while varying the external states of the environments without this affecting whether a subject experiences phenomenal attunement. Eva is, in other words, nothing like Pac-Man. One cannot simply screen-off as background conditions, her ongoing coupling to her cultural environment since this coupling is constitutive of her conscious experience of phenomenal lack of attunement. The idea that Eva can be unplugged from her surroundings so long as her internal states are kept the same relies on the idea that Eva’s experience is synchronically constituted. So long as you take two individuals who are internally the same at a given instant, this should necessitate that the individuals are also phenomenally identical. We take the failure of pluggability for the case of phenomenal attunement to follow from the diachronic constitution of phenomenal attunement. It is because phenomenal attunement is constituted by dynamical processes that interact over multiple timescales that individuals cannot simply be unplugged from one environment and plugged into another without this altering their experience of phenomenal attunement.

At this stage we anticipate some readers will raise the following worry: you state that it is not always possible to “unplug” the internal from the external without this having some non-trivial effect on phenomenal experience. But does the brain and its role in constituting consciousness not lend itself to this kind of “unplugging”? Think about cases such as dreaming, imagining, and mind wandering, in which the conscious mind is unplugged from the world, often allowing the brain to produce conscious states that are phenomenologically similar if not identical to those a subject enjoys when plugged into the world. We will call this the “internal fantasy objection” since it presses us to consider phenomenological similarities between perceptual experience and inner fantasies like dreaming and day-dreaming. If a subject can undergo a phenomenologically identical experience while being uncoupled from the world, doesn’t this undermine our claim that coupling is what constitutes phenomenal attunement? Couldn’t Eva dream she is back in Poland enjoying an experience of phenomenal attunement with her surroundings, while she is actually asleep in her bed in Vancouver?

Again this objection assumes Eva’s phenomenologically identical dream experience of attunement is the result of the brain states she undergoes at a moment in time. It assumes we can bracket Eva’s history of coupling to her environment and consider only what is happening in her brain at the moment she is dreaming as constitutive of her experience, treating everything else as a background condition. We suggest that dreaming and waking experiences can be phenomenologically indistinguishable because the neural processes that are necessary for waking experience are recycled in sleep. In online perceptual experience internal (brain) and external (world) states are tightly coupled to one another via sensory and active states (Kirchhoff and Kiverstein, 2019a,b). Break the coupling and you break the possibility for the system in question to constitute conscious experience of phenomenal attunement. Online experience is the result of a dynamical coupling of perceiver and environment that unfolds over time. In fully offline, decoupled cases of experience, such as in dreaming or mind-wandering, internal and external states are not coupled in the same way. But offline cases of conscious experience, insofar as they recycle online perceptual experience, remain *indirectly* constitutively dependent on a history of coupling. Offline experiences inherit this constitutive dependence on coupling from online experience insofar as they are the result of recycling online experiences. Perhaps it will be objected that the indirect constitutive dependence of dream experience on coupling is really just a causal dependence. But again, this response assumes that we can take the neural processes that are constitutive of dream experience at a moment in time and bracket the longer history of coupling with the environment. We’ve been arguing that such an assumption is false at least for the case of phenomenal attunement.^10^

The ever persistent internalist skeptic will no doubt continue to insist on the intuition that Eva^∗^ can have the same phenomenal experience as Eva, whatever differences there might be between their respective environments. The modal intuition is familiar: once we fix the neural contribution to consciousness, variation in the environment of the individual is beside the point. The phenomenal experience of Eva and Eva^∗^ is fully metaphysically determined by whatever is taking place within their brains. Eva^∗^ could just as well be a disembodied brain floating about in space (Block, 2005). All that matters when it comes to her phenomenal experience is the configuration of neural activity in her brain. We will call this the “free-floating brain” objection. We do not pretend to know what would happen in such remote possible worlds in which disembodied brains can suddenly spring into existence. There may be, at the outer limits of this modal intuition, a possible world where Eva and Eva^∗^ could share the same phenomenal experience despite living in different environments. What is of interest to us are possible worlds closer to home. We therefore suggest that the modal intuition that stands behind the free-floating brain objection is quite beside the point when it comes to the case of culture shock.

## Wide and Diachronic Constitution: Two Conceptual Flips

Internalist skeptics do not tire easily. We predict that they will continue to object, and most likely along quite familiar lines. It is something of a truism, they will insist, that cognitive activity (including conscious activity) is causally influenced by neural and non-neural (bodily, worldly) factors. But they will ask: How would we go about distinguishing non-neural bodily and worldly elements that are partially *constitutive* of the mind from such elements that merely *causal* influences on mental processes?

The causal-constitutive distinction has long dominated the debate about the extended mind (Adams and Aizawa, 2001, Adams and Aizawa, 2008; Rupert, 2004; Clark, 2008; Menary, 2010; Kirchhoff, 2015b; Kirchhoff and Kiverstein, 2019a). It will likely be objected that as defenders of ECM we are guilty of mistaking the causal dependence of phenomenal attunement on coupling with the cultural environment for the partial constitution of conscious experience by coupling with the cultural environment. Our opponents will assert that the proximal mechanisms internal to Eva’s brain are minimally sufficient for her conscious experience. Let us stipulate that the existence of a population of neurons *N* is minimally sufficient for a conscious experience *C* if the activation of *N* is all that is required for the generation of *C*. Other neural activity may be causally necessary for the subject to come to instantiate *N*, but once the subject is in neural state *N*, no other neural activity in addition to *N* is required for the subject to experience *C* (Hohwy and Bayne, 2015, p. 159).^11^ Our opponents will likely agree that the occurrence of *N* causally depends on a long prior history of engagement in cultural practices. Still they will say we must distinguish the proximate cause of Eva’s experience in the here and now—the configuration of neural states *N* that constitute the minimal sufficient condition for Eva’s experience—from whatever forms a part of the ultimate explanation for why Eva experiences what she does. It is only the proximate mechanisms that qualify as the realizers of her experience. The rest is a part of the ultimate explanation of why she has the experience she does. To insist otherwise is to commit the causal-constitutive fallacy.

This by now overly familiar line of argument is, in our view, premised on a number of problematic and mistaken assumptions. First, defenders of EM, most notably Clark and Chalmers (1998; but see also Wheeler, 2015), begin with the assumption that the basic ontological profile of the mind is a brainbound profile with the mind occasionally leaking out into the world. At least they assume that the brain plays a privileged role in constituting the mind. The paradigm of the mental is what goes on inside the head of individuals. The famous parity principle assesses the putative cognitive contribution of some external element by comparison with cognitive processes that take place internally inside of an individual’s head.^12^ This way of framing EM, however, concedes too much to the internalist, brainbound view of the mind. It assumes that the processes that take place inside of the brains of individuals are where constitutive causes are typically to be found. The external environment is populated with merely supporting causes, which may, under the right conditions, become constituent parts of a person’s mental states. This is to accept that the environment can basically be screened-off from constitutive questions about the mind by processes that are internal to individuals.^13^

Our argument for ECM does not rest on such an internalist starting point. As we said at the outset of this paper, we consider that mentality is first and foremost constituted by bouts of temporally extended engagement with the environment. The vast majority of “what humans do and experience is best understood by appealing to dynamically unfolding, situated embodied interactions and engagements with worldly offerings” (Hutto et al., 2014, p. 1). One cannot uncouple the cognitive agent from its cultural, developmental, and historical environment because much of what the agent does constitutively depends on his or her taking part in cultural practices. Our internalist opponents claim that external elements can only play supportive causal roles, but they do so because they start from the assumption that minds are typically housed inside of the skin and skull of individuals and only occasionally if ever have recourse to go out into the world. This is an assumption that internalists ironically share with first-wave parity-based arguments for the extended mind. We, by contrast, take this assumption to be precisely what EM ought to challenge.

The EM debate has up until now largely played out around the question of how to delineate the boundaries of mind. Philosophers have wondered how to settle the question of where the mind stops and the rest of the non-mental world begins, and the debate has ended up being all about “location, location, (and only) location” (Di Paolo, 2009, p. 10). The argument about the boundaries of the mind is, however, not only about the spatial location of the mind, and whether the constituents or material realizers of a given class of mental states are sometimes wide or always narrow. We have been making an argument for EM on temporal grounds because we take the constitution of mind to be diachronic, not synchronic. The focus on location has led to a reification of the proximate-ultimate distinction. Once we think of mind as diachronically constituted, a strict choice between proximate and ultimate explanation is revealed to be a *false choice*. There are no fixed and sharp boundaries between proximate and ultimate causes. Cultural practices and biological processes are best conceptualized as elements of a single dynamical network (cf. Hurley, 1998; Kirchhoff and Kiverstein, 2019a).

Consider once more the case of Eva and Eva^∗^. We argued that the main difference that determines why Eva^∗^ does, but Eva does not, experience culture shock is the coupling of the twins to the cultural environment. The constitution of Eva’s conscious experience of culture shock is not wholly determined by her properties as a biological individual at a given snapshot moment in time. It is the dynamics of her coupling with her cultural environment that pick out the constituents that make up the minimally sufficient constitutive basis of her experience of phenomenal lack of attunement characteristic of culture shock. Perception and action couple Eva to her cultural environment, but this coupling unfolds over time. The coupling is in turn constrained by patterns of practice that also unfold over longer periods of time. It is the meeting up of these temporally extended processes that constitutes the conditions under which Eva, but not Eva^∗^, experiences culture shock. So even when attempting to identify the minimally sufficient constitutive basis for certain kinds of conscious experience, one cannot simply separate the individual from her history. Constitution is not only wide; it is also *diachronic*.

The standard framing of the causal-constitutive distinction rests on a particular conception of the organism-environment relation; a conception according to which the world is “outside” or “external” to the organism and causes changes in its internal states. We have been arguing for ECM based upon the dynamics of the person’s coupling with her cultural environment in perception and action. The person is situated within a larger dynamical process of the cultural practices she takes part in (Kirchhoff et al., 2018). Culture is not something external to the individual in which the individual is sometimes causally embedded. Both the individual agent and the cultural environment form out of a nesting of dynamical networks, including networks that form in the patterns of activities people engage in over long periods of time as members of cultural practices. The cultural environment is not outside of the individual. The individual is situated in a cultural environment in a way that calls into question any neat distinction between inside and outside.

Even if the reader agrees with us on all of these points, she might still raise the following objection: if cultural practices, unfolding over longer than synchronic timescales, are partly constitutive of conscious experience, then there is no stopping the rampant and out of control expansion of the mind into the world. This is the well known cognitive bloat objection to EM (cf. Sprevak, 2009; Rowlands, 2010). The arguments of this paper provide us with resources for replying to this worry.

Consider again our twin case: Eva and Eva^∗^ are in identical neural states, but Eva is living in her home country, Poland, while Eva^∗^ has left to take up her new life in Vancouver. Eva^∗^, but not Eva, experiences culture shock. We have claimed the key difference is coupling to the cultural environment. Our appeal to an account that makes a difference in determining and differentiating constitutive causes from mere background causes allows us to sidestep the cognitive bloat objection. We have argued that the phenomenology of culture shock can be formalized as the Kullback–Leibler divergence between prior expectations (*P*^∗^) and cultural practices (*P*o) generating sensory states, *C*_exp_ = *D*_KL_ [*P*^∗^ || *P*o], such that high misalignment (i.e., high uncertainty) between *P*^∗^ and *P*o results in experiences of alienation and distress relative to current cultural practices. This leads to the following scenarios:

Poland: *C*_exp_ = *D*_KL_ [*P*^∗^ || *P*o] = 0. Here Eva’s expectations are aligned with her cultural world in such a fashion that she does not experience culture shock.
Vancouver: *C*_exp_ = *D*_KL_ [*P*^∗^ || *P*o] > 0. Here Eva’s expectations are misaligned with her cultural world in a way that results in her experiencing culture shock.

Counterfactually, were one to intervene in the cultural practices in Vancouver, one would expect a minimization in the divergence between *P*^∗^ and *P*o, with a resulting change in Eva’s phenomenology given the particular form of the agent-environment coupling. One might, for instance, point Eva to the district in Vancouver where a community of Polish emigres have made their home. Conversely, intervening in *P*^∗^ would likely lead to similar results, a reduced sense of distress and alienation. This provides our argument for ECM with a methodology for identifying relevant (i.e., constitutive) causes, demarcating these from mere background causes such as oxygen in the atmosphere, given that the latter would at best be an indirect (i.e., background) cause of the generation of conscious experience. There is therefore a path by which to argue for ECM that does not lead to unconstrained spreading consciousness out into the world.

## Conclusion

We have argued that the experience of phenomenal attunement is constituted by coupling to the cultural environment. A core structure of a person’s conscious mental life is constituted by processes that criss-cross the boundary separating the brain from the body and the rest of the world. We’ve made such an argument based on the diachronic constitution of phenomenal attunement. Many hold that the proximate-ultimate distinction marks a sharp divide between causes that track *why* a system does what it does and *how* a system is able to do what it does. This distinction is taken by most to represent a division between diachronic (ultimate) and synchronic (proximate) explanation. We have argued that this choice between two different modes of explanation is a false choice. An explanation of phenomenal attunement needs to embed ultimate causes (cultural practices and histories of engagement with the world) within a proximate explanation of conscious experience. This has led us also to call into question the distinction between causation and constitution as it is generally deployed in the EM debate, taking steps toward a diachronic conception of constitution. Diachronic constitution implies that the agent and the wider cultural environment cannot be cleanly unplugged from one another in a way that would allow for a purely neural (synchronic) explanation of phenomenal attunement. Conscious persons cannot simply throw away the world and rely wholly on on-board neural resources for the generation of their conscious experience of being attuned to the world. Conscious beings cannot be unplugged from the extended dynamic singularity that forms in the agent’s coupling with the world because conscious beings are extended dynamic singularities.

## Author Contributions

MK and JK have contributed equally to the production of this research article and approved the submitted version.

## Conflict of Interest

The authors declare that the research was conducted in the absence of any commercial or financial relationships that could be construed as a potential conflict of interest.

## Footnotes

1 Others have defended positions on consciousness closely related to the extended mind view we develop in this paper. Ward (2012), for example, does so by appealing to personal-level considerations about the phenomenology of experience and what such considerations might tell us about the sub-personal level machinery involved in the constitution of consciousness. Others, like Noë (2004, 2009), appeal to active sensorimotor engagement with the environment to make their case (see also Hurley, 1998; Rowlands, 2010). Cosmelli and Thompson (2010) have argued for the constitutive dependence of consciousness on the non-neural body and world by calling into question brain-in-vat intuitions. Northoff (2019) can also be read as arguing for an account of consciousness as partially constituted by factors beyond the brain. Our argument will, however, differ from these important exponents of ECM. We argue for the constitutive dependence of conscious experience on cycles of perception and action that couple a person to his or her cultural environment. We base our argument for the extendedness of phenomenal attunement on a diachronic account of constitution.

2 KL-divergence is a part of the formal tools used for modeling consciousness in terms of belief or expectation updating schemes such as predictive processing in cognitive neuroscience (Hohwy, 2012; Clark, 2016; Friston, 2018; Kirchhoff and Kiverstein, 2019a). It might be objected that this alignment between *P*^∗^ and sensory states with cultural practices can be explained from entirely inside of the brain. The remainder of our paper is devoted to explaining why we think such an objection is mistaken. See also Northoff (2019) for an account of why KL-divergence (which he understands in terms of variational free energy) is best interpreted in environment-involving terms (and Kirchhoff and Kiverstein, 2019a for a book-length treatment of the extended conscious mind cast in terms of active inference and predictive processing).

3 In neural dynamical terms, we can think of attunement with respect to generalized synchrony of the large-scale internal dynamics of the brain with the external dynamics of the cultural environment. A simple example of generalized synchrony is entrainment of the sort that happens when one finds one tapping one’s foot along to the rhythm of music one is listening to. Northoff (2019) suggests that in perception the temporal-spatial dynamics of the brain synchronize with, and thus conform to, the world’s external dynamics, while in action it is the other way around: the world’s external dynamics conforms to the brain’s internal dynamics (c.f. Bruineberg and Rietveld, 2014; Bruineberg et al., 2018; Kirchhoff and Kiverstein, 2019a, ch. 4). Northoff makes a more general argument for ECM on the basis of these kinds of considerations. We restrict our focus here to arguing for the extension of phenomenal attunement.

4 Krueger and Maiese (2018, p. 28) have noted that a key challenge people on the autism spectrum face is becoming attuned to the norms and expectations of non-autistic “neurotypicals,” which are often “unspoken, highly context-specific and communicated by way of nuanced body language.” They go on to write that “high-functioning” people with autism often find it easy to become attuned to each other since their interactions are governed by “autism-friendly” norms and expectations.

5 Note, we are not questioning that problem solving may take this form, assembled and unfolding over synchronic timescales in the here and now. The point we are objecting to is that by limiting the assembly of even extended minds to synchronic timescales, one thereby rules out, unnecessarily, cultural practices unfolding over longer timescales from playing a role in the material constitution of what people say and do. A core aspect of our argument for ECM is precisely to question this privileging of the synchronic timescale.

6 Our thanks to the reviewer for pressing this objection.

7 Note that even answering this *how*-question presupposes a diachronic notion of constitution, for the processes involved in enabling flight are themselves temporally extended processes. To say that a system is in some particular state *X* at a particular point in time is to say that the average of the system’s states during that period of time was *X* (see Spivey, 2007, for discussion). Thus, we do not agree that *how*-explanations must always posit mechanisms whose workings are synchronic. We are suggesting that such an assumption does not hold in the case of dynamical phenomena.

8 One might worry that our account inherits the problem of how to delineate between mere ultimate causes and ultimate causes as constituent causes. We return to this worry below in section “Wide and Diachronic Constitution: Two Conceptual Flips.”

9 The cognitive neuroscientist Georg Northoff (2019) would seem to agree. He argues that “what happens beyond the boundaries of our brain” in the body and in the surrounding environment is partially constitutive of consciousness (Northoff, 2019, p. 12). The brain’s intrinsic dynamics should, he suggests, be thought of as “neuro-ecological” —that is to say, as “deeply embedded within and dependent upon the world” (Northoff, 2019, p. 7). We take this concept to be another way of talking about the unpluggability of the brain from the body and the rest of the world.

10 Our thanks to the reviewer for helpful discussion of this point.

11 Notice that this characterization of minimal neural sufficiency is very general. It is neutral on debates within the neuroscience of consciousness about the best theory of consciousness, such as the debate between the information integration theory of Tononi and colleagues, the global workspace theory of Baars and Dehaene, or the recurrent processing theory of Lamme and colleagues, to mention a few candidates. Our discussion need not take a stand on which of these theories is correct since we are concerned with the more general question of the correctness of the neural sufficiency claim.

12 For example, in their discussion of Otto, Clark and Chalmers (1998) argue that the inscriptions in Otto’s notebook are part and parcel of Otto’s mind conditioned on an appeal to the function of brain-based biological memory. In a different example also considered by Clark and Chalmers (1998), the comparison is hypothetical, in the consideration of different instantiations of the function of a zoid-rotator in the game of Tetris. In both scenarios, however, it is the internal that is paraded as the benchmark for the mental. With this starting point in place, the real business of EM is to test whether specific external elements in the world play comparable or equivalent functional roles to those identified inside the head. If yes, then the external elements in question should fall within the confines of the mind.

13 The notion of “screening-off” is standard in discussions of mental causation. As we pointed out in section “Synchronic and Diachronic Constitution,” it is common procedure to look for causes that play the most salient role in the production of some phenomenon when identifying constituents of such a phenomenon.
